# Supplementary material for: Ontogeny of long-range vocalizations in a Neotropical fossorial rodent: the Anillaco Tuco-Tuco (Ctenomys sp.)
Source: PeerJ. 2018 Feb 14;6:e4334. doi: 10.7717/peerj.4334 (PMC5816585; doi:10.7717/peerj.4334)
Supplement: Table S1 — Acoustic parameters (duration 90%, bandwidth 90%, and peak frequency) of triad notes and individual notes of four different age classes (days after birth) of juvenile and adult males (n = 5) and females (n = 3) of the Anillaco Tuco-Tuco (Ctenomys sp.). Data presented is mean ± sd (range) (sample size). [file peerj-06-4334-s001.docx]

| **Parameter** |  | **Age classes (days after birth)** | **Juveniles** | | | | | | | | **Adults** | |
| --- | --- | --- | --- | --- | --- | --- | --- | --- | --- | --- | --- | --- |
|  |  |  | **Male 1** | **Male 2** | **Male 3** | **Male 4** | **Male 5** | **Female 1** | **Female 2** | **Female 3** | **Males** | **Females** |
| **Duration 90% (ms)** | **Series** | **14–28** | 29.5±2.9  [26.1-31.9]  (2) | 25.6±2.2  [23.2- 29.0]  (2) | 26.8±2.0  [24.0-29.3]  (2) | 29.9±3.5  [26.6-37.8]  (3) | 26.1±1.4  [24.0-26.7]  (1) | 26.6±2.8  [23.2-31.9]  (2) | 25.6±2.1  [23.2-29.0]  (2) | - | 31.4±1.7  [29.3-34.6]  (5) | 31.3±0.1  [29.3-32.0]  (3) |
|  |  | **44–58** | 33.4±1.6  [31.9-34.8]  (2) | 28.4±3.2  [23.2-31.9]  (3) | 29.8±2.0  [26.7-32.0]  (2) | 30.8±3.5  [26.6-34.6]  (3) | 30.2±3.2  [26.7-34.7]  (2) | 30.6±1.5  [29.0-31.9]  (3) | 28.0±3.5  [23.2-32.0]  (2) | - | - | - |
|  |  | **74–88** | 33.2±2.6  [29.0-37.7]  (3) | 30.3±1.5  [29.0-31.9]  (3) | 32.9±2.4  [29.0-34.8]  (2) | 31.4±2.9  [26.6-34.7]  (3) | 32.6±2.4  [29.6-37.7]  (3) | 32.2±2.2  [29.2-37.7]  (3) | 30.9±1.5  [29.0-32.0]  (2) | - | - | - |
|  |  | **104–118**  **104–118** | 30.3±2.6  [26.1-34.8]  (3) | 31.9±2.5  [29.0-34.8]  (3) | † | 31.9±1.8  [29.0-34.8]  (2) | 30.6±3.9  [26.2-34.8]  (3) | 30.5±1.5  [29.0-31.9]  (2) | 32.2±2.0  [29.0-34.8]  (3) | - | - | - |
|  | **Individual notes** |  | 31.9±0.0  [31.9-31.9]  (3) | 32.9±1.7  [31.9-34.8]  (3) | † | 31.9±0.0  [31.9-31.9]  (3) | - | - | - | - | 32.2±2.0  [29.3-34.6]  (15) | 30.2±1.5  [29.3-32.0]  (9) |
| **Bandwidth 90% (Hz )** | **Series** | **14–28** | 509.6±69.9  [430.7-624.5]  (2) | 2065.4±1159.8  [1367.4-4,414]  (2) | 908.5±308.1  [574.2-1,441.4]  (2) | 259.9±26.0  [222.7-290.7]  (3) | 984.3±435.4  [574.2-1,441]  (1) | 2,083.3±288.9  [1,733-2,476]  (2) | 1008.5±714.6  [430.7-2067]  (2) | - | 192.7±67.8  [105.5-257.8]  (5) | 293.0±57.4  [246.1-363.3]  (3) |
|  |  | **44–58** | 504.2±202.0  [333.8-807.5]  (2) | 637.6±137.1  [452.2-786.0]  (3) | 494.1±136.1  [351.6-738.3]  (2) | 235.6±70.2  [172.3-376.8]  (3) | 212.8±66.4  [128.9-269.5]  (2) | 521.5±93.4  [312.2-613.7]  (3) | 464.2±117.1  [363.3-678.3]  (2) | - | - | - |
|  |  | **74–88** | 301.4±74.7  [215.3-398.4]  (3) | 294.3±114.5  [226.1-592.2]  (3) | 206.3±31.5  [161.5-247.6]  (2) | 218.7±80.0  [164.1-421.9]  (3) | 230.8±71.8  [96.9-355.3]  (3) | 308.0±153.4  [150-678.3]  (3) | 439.0±70.9  [363.3-550.8]  (2) | - | - | - |
|  |  | **104–118**  **104–118** | 174.6±101.9  [86.1-355.3]  (3) | 258.3±44.7  [204.6-312.2]  (3) | † | 199.2±23.4  [175.8-222.7]  (2) | 199.7±79.8  [86.1-312.2]  (3) | 213.2±60.2  [140.3-279.6]  (2) | 296.6±79.1  [183.0-398.4]  (3) | - | - | - |
|  | **Individual notes** |  | 312.2±28.5  [279.9-333.8]  (3) | 326.6±32.8  [290.7-355.3]  (3) | † | - | - | - | - | - | 295.6±60.5  [234.4-375.0]  (15) | 371.0±112.0  [257.8-492.2]  (9) |
| **Peak frequency**  **(Hz)** | **Series** | **14–28** | 222.5±26.9  [193.8-258.4]  (2) | 445.0±179.5  [279.9-646.0]  (2) | 596.0±289.9  [234.4-1,033.6]  (2) | 291.6±10.8  [281.2-316.4]  (3) | 359.4±108.8  [234.4-433.6]  (1) | 606.5±173.2  [463.0-893.6]  (2) | 410.9±115.6  [333.8-570.6]  (2) | - | 187.5±15.6  [164.1-210.9]  (5) | 249.0±17.5  [234.4-269.5]  (3) |
|  |  | **44–58** | 215.3±122.2  [118.4-376.8]  (2) | 215.3±25.2  [183.0-258.4]  (3) | 306.6±174.6  [140.6-503.9]  (2) | 257.8±11.7  [234.4-269.5]  (3) | 294.9±11.5  [281.2-304.7]  (2) | 339.0±20.9  [312.5-376.8]  (3) | 299.8±39.3  [246.1-344.5]  (2) | - | - | - |
|  |  | **74–88** | 190.2±65.4  [107.7-301.5]  (3) | 235.7±37.9  [161.5-301.5]  (3) | 217.1±35.6  [150.7-247.7]  (2) | 263.1±24.1  [236.9-312.2]  (3) | 180.6±65.4  [118.4-344.5]  (3) | 339.7±130.9  [161.5-549.1]  (3) | 249.5±20.9  [226.1-279.9]  (2) | - | - | - |
|  |  | **104–118**  **104–118** | 168.6±29.4  [140.0-236.9]  (3) | 189.0±37.3  [150.7-236.9]  (3) | † | 218.9±81.3  [140.0-366.1]  (2) | 160.3±23.7  [118.4-193.8]  (3) | 324.7±12.5  [312.2-344.5]  (2) | 229.0±31.6  [187.5-281.2]  (3) | - | - | - |
|  | **Individual notes** |  | 154.3±6.2  [150.7-161.5]  (3) | 195.3±59.5  [140.0-258.4]  (3) | † | 140.0±0  [140.0-140.0]  (3) | - | - | - | - | 166.7±14.0  [152.3-187.5]  (15) | 244.1±15.5  [222.7-257.8]  (9) |
